# Carmi Syndrome in a Preterm Neonate: A Multidisciplinary Approach and Ethical Challenge

**DOI:** 10.1155/2018/4548194

**Published:** 2018-12-20

**Authors:** Timothy D. Hicks, Himanshu Singh, Michel Mikhael, Anita R. Shah

**Affiliations:** Department of Neonatology, Children's Hospital of Orange County, Orange, CA, USA

## Abstract

Epidermolysis bullosa (EB) is characterized by blistering of the skin and mucosal erosions caused by hemidesmosomal abnormalities. EB is divided into 3 major subgroups depending on the particular location of tissue separation: EB simplex, dystrophic EB, and junctional EB. Junctional EB (JEB) can further be broken down into Herlitz, non-Herlitz, and JEB with pyloric atresia (Carmi syndrome) depending on genetic and histologic testing. When extensive, management of a patient with EB can be challenging due to not only cutaneous but also extracutaneous manifestations as well. Families and health care teams are often faced with difficult decisions in their infant's best interest. We report a case of a preterm neonate with Carmi syndrome and unique findings on immunofluorescence studies. The patient's course was complicated by multisystem involvement and ultimately death. A multidisciplinary approach was crucial in the light of diagnostic, therapeutic, and ethical challenges.

## 1. Introduction

Epidermolysis bullosa (EB) is an inherited, autosomal recessive, bullous disease, characterized by mucosal erosions and skin blisters. Numerous subtypes of EB are described and are divided into three major groups: EB simplex (EBS), dystrophic EB (DEB), and junctional EB (JEB). Junctional EB is further divided into three subgroups: Herlitz, non-Herlitz, and JEB with pyloric atresia (JEB-PA) also known as Carmi syndrome. JEB has a proven familial predisposition and is caused by mutations in the ITGA6, ITGB4, LAMA3, LAMB3, LAMC2, and COL17A1 genes [1]. Patients with Herlitz JEB have the poorest prognosis with an estimated mortality rate approaching 90% during the first year of life [2]. Patients with JEB-PA also have a poor prognosis with a mortality rate up to 75% due most commonly to sepsis and renal failure [3].

## 2. Case Report

**Figure 1:**
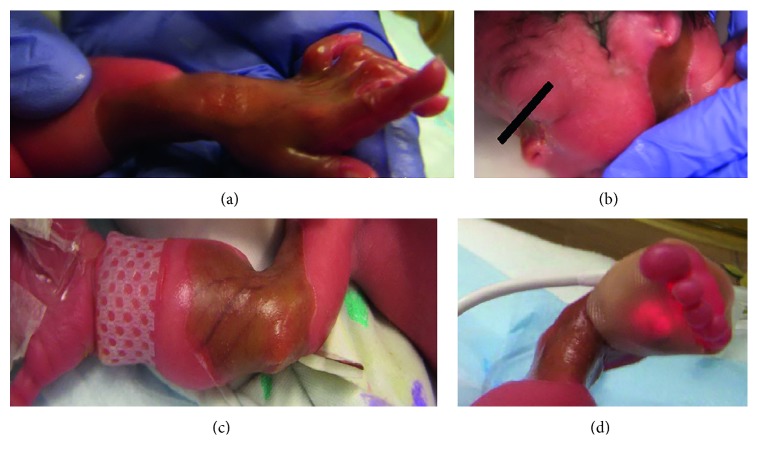
Cutaneous involvement of the neonate. (a) Involvement of the left wrist and hand. (b) Absence of skin on the nasal bridge and neck. Microtia also notable. (c) Involvement of the right forearm and elbow. (d) Circumferential epidermolytic lesions on right ankle.

A 30-week gestation male was born via vaginal delivery to a G2P2 mother in a nonconsanguineous marriage with appropriate prenatal care and an unremarkable prenatal course. The premature delivery was precipitated by placental abruption and preterm onset of labor. The mother received two doses of antenatal steroids prior to delivery. Apgar scores were 8 and 9 at 1 and 5 minutes of life, respectively. On examination, the baby was noted to have bilateral microtia along with extensive truncal and appendicular epidermolytic lesions (Figure 1).

The infant was started on prophylactic ampicillin and gentamicin given his prematurity and open skin wounds. Aquaphor barrier cream and soft silicone dressings were applied, and the patient was placed in reverse isolation. He was placed in a 34°C temperature-controlled incubator without humidity, as heat and humidity can exacerbate blistering. Dermatology, plastic surgery, wound care, and genetics services were consulted upon the patient's admission.

**Figure 2:**
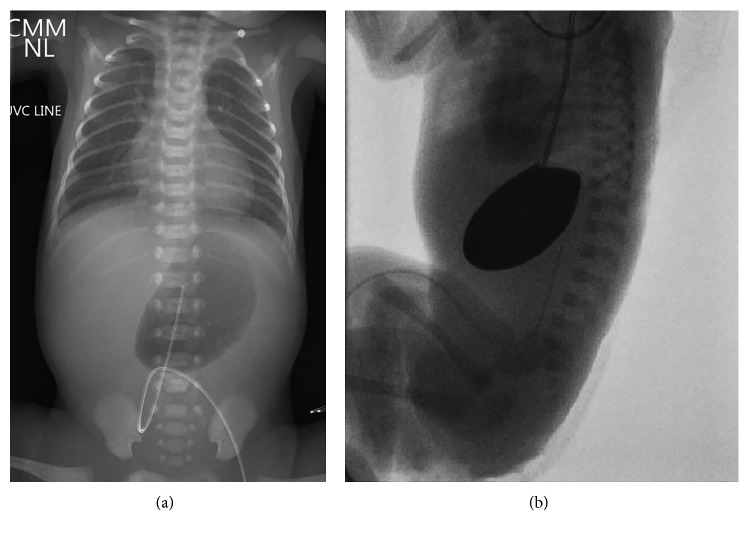
Radiographic imaging consistent with pyloric atresia. (a) Abdominal X-ray showing gaseous distension of the stomach with no free intra-abdominal air. (b) Fluoroscopic abdominal X-ray showing total gastric outlet obstruction with no contrast entering the duodenum.

On the first day of life, he had numerous desaturation episodes likely secondary to prematurity in conjunction with sloughing of the respiratory epithelium. He was intubated and placed on mechanical ventilation support. High gastric output was noted without abdominal distension, and a combined chest and abdominal X-ray showed mild gaseous distension of the stomach with no free intra-abdominal air (Figure 2). An orogastric tube was placed to low intermittent suction, and an upper gastrointestinal tract fluoroscopic examination was performed revealing a complete discontinuity between the stomach and duodenum, consistent with pyloric atresia (Figure 2).

Surgical repair was tentatively scheduled with concerns around the adequacy of postoperative healing. A screening renal ultrasound showed a right multicystic and dysplastic kidney. On the second day of life, a skin biopsy of the left upper extremity was obtained by dermatology. Renal function was followed closely, and medication dosage was renally dose adjusted. Gentamicin was switched to cefotaxime to avoid nephrotoxic side effects. By the sixth day of life, the patient's creatinine had increased to 1.6 mg/dL. On day of life 7, a decision was made to discontinue ampicillin and cefotaxime after blood cultures remained negative. Bacitracin ointment was started topically. After 12 hours without antibiotics, the patient was febrile to 38.4°C; after blood and urine cultures were sent, piperacillin-tazobactam and oxacillin were started. The white blood cell count was noted to be 13.4·10^9^/L with 18% neutrophils and 14% bands. During the urinary bladder catheterization, red discoloration of the urine was noted. The blood and urine cultures had no growth at 48 hours.

**Figure 3:**
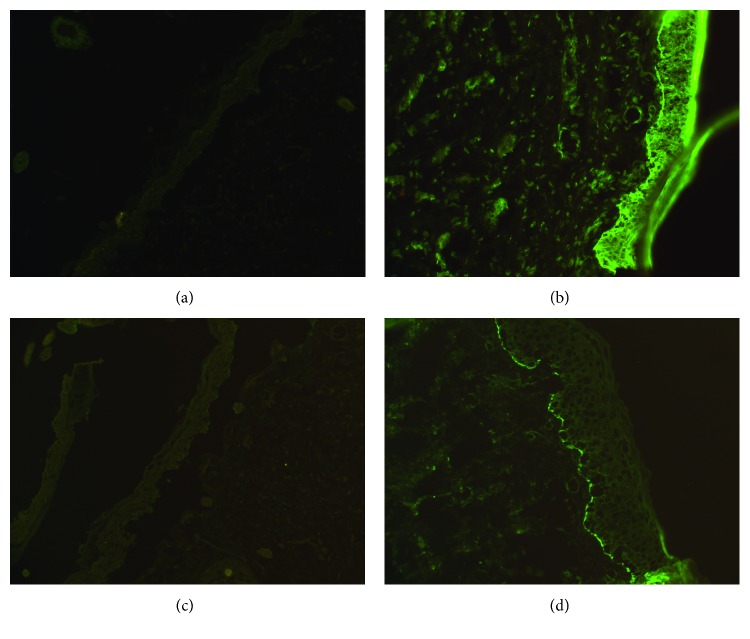
Immunofluorescence staining. (a) Absence of *α*6 integrin staining compared to the control (b). (c) Absence of *β*4 integrin staining compared to the control (d).

On the eighth day of life, the pathology report using immunofluorescence mapping showed an absence of *α*6-*β*4-integrin consistent with JEB-PA, Carmi syndrome. Incomplete staining on laminin-332 (formerly laminin-5) was also noted (Figure 3).

Simultaneously, the infant's renal function continued to worsen with serum creatinine levels rising to 2.0 mg/dL and serum sodium decreasing to 131 mEq/L, so the nephrology team was consulted. They recommended increasing total fluid intake due to possible prerenal factors contributing to the acute kidney injury in light of insensible water losses due to JEB. The following day, the serum sodium decreased further to 122 mEq/L, so fluids were restricted which showed improvement in the sodium level. On day of life 10, while still on antibiotics, the patient was again febrile to 38.7°C. Two wound cultures were obtained, another blood culture was sent, and labs showed WBCs of 29.7·10^9^/L with 50% neutrophils and 8% bands. The blood culture was again negative at 48 hours. One of the wound cultures grew coagulase negative *Staphylococcus* while the other was negative. After consulting with the infectious disease team, antibiotics were discontinued since there was no obvious source of infection. On day of life 11, the infant's renal function worsened with a creatinine of 3.6 mg/dL. Fluids were liberalized to protect his kidney function while closely watching for reoccurring hyponatremia. Gross hematuria then developed with a further increase in the creatinine level to 5.1 mEq/L.

A multidisciplinary meeting took place involving the mother and her partner, the attending neonatologist, geneticist, nephrologist, palliative care, and ancillary staff. After gleaning the mother's understanding of her infant's condition, the medical team clarified the grave prognosis of Carmi syndrome compounded by renal failure. After a discussion of goals of care, a range of compassionate options was presented to the family. One possibility was aggressive medical management including possible dialysis and surgical correction of the infant's pyloric atresia. Due to the extent of blistering, the uncertainties of meaningful wound healing from a dialysis catheter placement, surgical skin incisions, intestinal re-anastomosis, and the potential increased risk of infection were presented to the family. Other options included a redirection of care towards comfort: limiting aggressive medical interventions with the understanding that the infant may not survive. In our specific case, when presented with a range of compassionate options, this family held the best interests of their child at the core of their decision-making, valuing comfort. The medical team, respecting this decision as well as upholding principles of nonmaleficence agreed to comfort care. The family opted for palliative care with compassionate extubation allowing them to hold the infant. Skin care, intravenous fluids, and pain medication were continued for comfort. Once the extended family was at the bedside, the infant's respiratory support was removed and the baby died 48 hours later, on day of life 17.

## 3. Discussion

Swinburne and Kohler first described the association between PA and EB in 1967 at the 13^th^ Annual Pediatric Pathology Society in England and published the corresponding paper in 1968 [4]. In 1982, Carmi described 2 cases of aplasia cutis congenita (ACC); one was extensive and was associated with pyloric atresia [5]. A year later, the eponym Carmi syndrome emerged in literature [6]. Both PA and EB are rare, autosomal recessive entities; however, the combination of these diseases appears more than coincidental [7]. The exact prevalence of the subtype JEB-PA is unknown, but thought to be <1 : 1,000,000. About 100 cases have been documented worldwide [8].

Prenatally, JEB-PA can present with polyhydramnios or fetal gastric dilation as seen on ultrasound. For pregnancies at risk, prenatal diagnosis can be established by examining DNA from chorionic villi or amniotic fluid cells or by using monoclonal antibodies. Additionally, direct skin biopsies can be obtained via fetoscopy or ultrasound guidance to confirm the diagnosis [9, 10]. The majority of neonates are born prematurely [11].

Clinical features may be apparent at birth or may appear later. The skin may show localized or extensive areas of peeling or blistering with little or no trauma. Significant oral and mucous membrane involvement is common. Additional features of EB-PA may include fusion of the skin between the fingers and toes, nail dystrophy, scarring alopecia, contractures, and dilated cardiomyopathy. PA is usually suspected when neonates develop recurrent non-bilious vomiting and abdominal distension.

**Figure 4:**
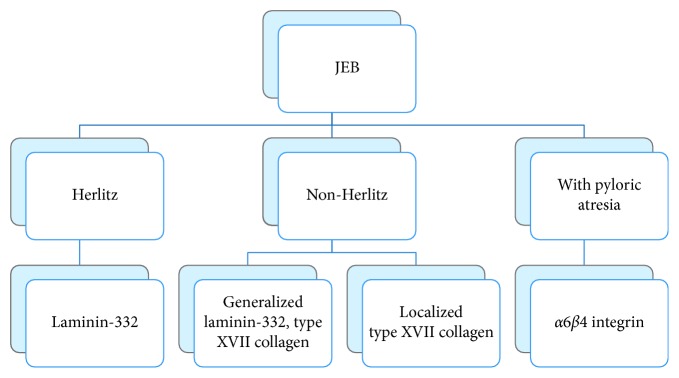
JEB subtypes and the protein abnormalities.

Diagnosis is usually suspected based on clinical findings of skin blistering and gastric outlet obstruction. The most precise means of diagnosing inherited EB involves the assessment of a combination of ultrastructural and antigenic features by transmission electron microscopy, immunofluorescence antigenic mapping, and EB-related monoclonal antibody studies. Molecular genetics are available but not necessary for diagnostic confirmation. Depending on the level of basement membrane cleavage, three major varieties of EB have been identified: simplex EB (epidermolytic), junctional EB (lucidolytic), and dystrophic EB (dermolytic). All three types of EB have been described to coexist with congenital PA. As previously mentioned, JEB is further broken down into 3 main categories: JEB Herlitz (JEB-H), JEB non-Herlitz (JEB-nH), and JEB with pyloric atresia (JEB-PA). JEB-H is the most severe with widespread lesions and death early in the neonatal period, associated with absent laminin-332 (formerly laminin-5) on immunofluorescence (IF) studies. JEB-nH can be broken down into generalized and localized depending on the amount of lucidolytic and extracutaneous involvement. Generalized JEB-nH stains incompletely with laminin-322 and type XVII collagen. Localized JEB-nH stains incompletely with type XVII collagen. Lastly, JEB-PA stains with absent or severely decreased *α*6*β*4 integrin [1] (Figure 4).

Uniquely, our patient had absent *α*6*β*4 integrin along with altered laminin-322 staining. Perhaps, the abnormal laminin-322 protein directly led to the severity of skin lesions; since, when absent, it leads to the most severe Herlitz presentation. Chang et al. proposed that PA is secondary to an intrauterine complication of EB in which the sloughing pyloric mucosa leads to fibrosis and obstruction of the pyloric canal [12]. However, Lestringant et al. thought that PA was the pleiotropic effect of a single gene [11]. Atresia involving multiple regions of GI tract may occur simultaneously, such as esophageal and anal atresia [13].

Uroepithelium similar to other epithelialized structures may be affected by EB, as seen in our patient [14]. In particular, genitourinary malformations such as dysplastic and multicystic kidneys, hydronephrosis, hydroureter, ureterocele, duplicated renal collecting system, and absent bladder are common as extensive injury and fibrosis of the uroepithelium can lead to obstructive uropathies [15]. Evaluation for possible uropathy is crucial for patients with EB. In 2018, Mylonas et al. conducted the first systematic literature review on Carmi syndrome. The clinical data, histological findings, and outcomes of 100 previously reported cases were analyzed. Of these, 11% had renal failure [3]. For our patient, in addition to the unusual histopathology, the infant also suffered from renal failure, which heightened the complexity of medical management and decision-making.

There is no definitive treatment for JEB-PA. If a prenatal diagnosis is established, a caesarean section should be considered to reduce trauma during delivery. Postnatally, the patient should be immediately placed in contact isolation. Dressings and ointments should be used to cover open blisters with wound care or plastic surgery consultation. Ointments can also be used to cover gloves and medical equipment to avoid skin damage, and specialized tape should be considered when securing vascular access devices. Environmental humidity and heat need to be closely regulated. Supportive measures including antibiotics, adequate hydration, and nutritional support enhance care. Prevention of secondary complications such as growth delay, anemia, zinc deficiency, osteopenia/osteoporosis, and scarring of skin and mucosal surfaces are best addressed in a multidisciplinary fashion. A timely plain abdominal radiograph needs to be performed in all babies with EB to exclude PA. Surgical intervention may be indicated to correct PA. While the surgical management for PA is straightforward, poor wound healing with possible infections, protein loss, and failure to thrive complicate the severe lucidolytic skin lesions, often leading to death. Previously, this has led to the recommendation that surgical treatment be withheld in patients with EB-PA [16]. However, a series by Hayashi et al. reported long-term survival in 4 of 5 patients encouraging aggressive management in affected neonates, especially if patients are stable [17, 18]. Contrastingly, the systematic review by Mylonas et al. showed worse outcomes; 73 patients out of 100 with JEB-PA cases underwent a PA repair and 49 (67.1%) died [3]. Currently, clear-cut treatment recommendations remain elusive, thus shared decision-making between the parents and the care team is of utmost importance. Parents are afforded a unique position in society to make decisions for their neonates, as infants cannot consent for themselves, and at the core of this type of surrogate decision-making must be the best interests of the neonate [19].

In many cases, the course of JEB-PA is relentless and fatal despite surgical correction of concomitant PA. The prognosis of this disease is poor due to prematurity, extensive skin blistering with fluid and electrolyte imbalance, respiratory morbidities, malnutrition, sepsis, and associated significant renal and genitourinary disease. Of the 100 reported cases as of 2018, 94 included survival status and of those 70 died (74.5%) with median time of death 30 days of age [3]. In those who survived, the condition sometimes improved with time, with some patients seeing improved blistering later in life. However, many affected individuals living past infancy experience blistering and formation of granulation tissue around the mouth, nose, fingers and toes, and occasionally in the trachea, leading to stridor. The different JEB subtypes and the specific protein abnormalities may explain this variation in survival. Thus, an exact diagnosis of EB subtype may be a useful indicator of prognosis helping medical teams and families in difficult decision-making [16, 20].

With this case, we presented a severe generalized form of JEB-PA with unique histopathology: absent *α*6*β*4 integrin and altered laminin-322. The infant's course was unusually complicated by multisystem involvement including renal failure. We hope this discussion helps clinicians better understand Carmi syndrome and its complications, both medical and ethical.
